# Consequences of Global Warming of 1.5 °C and 2 °C for Regional Temperature and Precipitation Changes in the Contiguous United States

**DOI:** 10.1371/journal.pone.0168697

**Published:** 2017-01-11

**Authors:** Ambarish V. Karmalkar, Raymond S. Bradley

**Affiliations:** Northeast Climate Science Center and Climate System Research Center, Department of Geosciences, University of Massachusetts Amherst, Amherst, Massachusetts, United States of America; Universidade de Vigo, SPAIN

## Abstract

The differential warming of land and ocean leads to many continental regions in the Northern Hemisphere warming at rates higher than the global mean temperature. Adaptation and conservation efforts will, therefore, benefit from understanding regional consequences of limiting the global mean temperature increase to well below 2°C above pre-industrial levels, a limit agreed upon at the United Nations Climate Summit in Paris in December 2015. Here, we analyze climate model simulations from the Coupled Model Intercomparison Project Phase 5 (CMIP5) to determine the timing and magnitude of regional temperature and precipitation changes across the contiguous United States (US) for global warming of 1.5 and 2°C and highlight consensus and uncertainties in model projections and their implications for making decisions. The regional warming rates differ considerably across the contiguous US, but all regions are projected to reach 2°C about 10-20 years before the global mean temperature. Although there is uncertainty in the timing of exactly when the 1.5 and 2°C thresholds will be crossed regionally, over 80% of the models project at least 2°C warming by 2050 for all regions for the high emissions scenario. This threshold-based approach also highlights regional variations in the rate of warming across the US. The fastest warming region in the contiguous US is the Northeast, which is projected to warm by 3°C when global warming reaches 2°C. The signal-to-noise ratio calculations indicate that the regional warming estimates remain outside the envelope of uncertainty throughout the twenty-first century, making them potentially useful to planners. The regional precipitation projections for global warming of 1.5°C and 2°C are uncertain, but the eastern US is projected to experience wetter winters and the Great Plains and the Northwest US are projected to experience drier summers in the future. The impact of different scenarios on regional precipitation projections is negligible throughout the twenty-first century compared to uncertainties associated with internal variability and model diversity.

## Introduction

The world leaders gathered for the 21^st^ Conference of the Parties (COP21) in Paris in December 2015 agreed to take steps towards limiting the global mean annual surface air temperature (GMAT) increase to well below 2°C above pre-industrial levels, and to pursue efforts towards a target of 1.5°C [1]. Many studies argue that the 2°C target is overly optimistic [2, 3], since the global emissions are currently tracking the high end of plausible scenarios [4, 5] resulting in stringent limits on cumulative CO_2_ emissions [6]. While there is no real scientific basis to why global warming of 2°C should be considered ‘safe’ [7], it emerged as “the least unattractive course of action” [8] and has been used as an easily understood, politically useful marker to communicate the urgency of the climate change problem and to drive action on a global scale. The inclusion of 1.5°C in the Paris Agreement, initially proposed by the small island nations, is especially pertinent to avoid the worst impacts of rising sea levels, and declining Arctic sea ice among other factors [9]. Unlike 2°C, the 1.5°C target is relatively unexplored, and studies highlighting its feasibility and impacts are beginning to emerge [10–13]. Under most low emission scenarios, global warming overshoots 1.5°C some time during the 21^st^ century before dropping to this limit around or after 2100 [11]. As a result, 1.5°C is considered an aspirational ‘long-term’ temperature goal.

The GMAT increase of 1.5 to 2°C—relevant for global scale policy recommendations and mitigation strategies—is not a helpful metric for impacts assessment and adaptation planning at regional scales. The land regions of the Northern Hemisphere are warming at rates faster than the globe [14] and are projected to cross the 2°C target before GMAT [15]. Therefore, information on regional climate projections and associated uncertainties for global warming thresholds of 1.5 and 2°C can prove beneficial to effectively communicate regional consequence of the Paris Agreement to the regional stakeholders (e.g., hydrologists, ecologists, resource planners). A study based on the Coupled Model Intercomparison Project Phase 3 (CMIP3) simulations [15] demonstrated that the contiguous United States (CONUS) is projected to experience 2°C warming during the 2040s under the A1B scenario (see Fig 4 in [15]). Their analysis focussed on regions at subcontinental scales and is inadequate to determine how the rate of warming varies across the country, which is more relevant for regional planning. The historical warming trends are different across the country with the northern and Southwest US warming at much faster rates than the Southeast and southern Great Plains [16]. Similarly, the observed precipitation has increased over CONUS in the 20^th^ century, but with substantial seasonal and regional differences [16]. Studies of climate projections [16, 17], however, do not discuss how projected warming trends vary across the US. Also, the traditional approach of using multi-model means for a future 20-30 year period to present climate change information is not useful in communicating the timing of regional changes for specific thresholds. We use data from the CMIP5 [18] multi-model ensemble (MME) for two Representative Concentration Pathways (RCPs [19]) to compare global, national and regional projections in terms of the timing and magnitude of temperature and precipitation changes for different global warming thresholds. Specifically, we provide a regional perspective for COP21 global temperature targets by addressing the following questions: (i) when will 1.5 and 2°C warming occur regionally in the US? and (ii) what are the temperature and precipitation projections for a region when GMAT crosses 1.5 and 2°C warming thresholds? Furthermore, we also describe agreement and uncertainties in model projections.

## Data and Methods

The analysis is based on climate model simulations that contributed to the fifth phase of the Coupled Model Intercomparison Project (CMIP5 [18]). The CMIP5 MME used here comprises 32 models (S1 Text) and includes surface air temperature (S1 File) and precipitation data (S2 File) from historical and two RCP (RCP4.5, RCP8.5) simulations. The simulations over the historical period (1880-2005) are compared against gridded observations: Climate Research Unit Time Series (CRU TS version 3.23) data for temperature [20] and University of Delaware (UDel) data for precipitation [21]. All model data and observations were first regridded to a common 2.5°×2.5° latitude-longitude grid using bilinear interpolation to allow for comparisons to be made at same resolution. Temperature and precipitation anomalies were calculated relative to the mean of 1901-1930, the period which is common to all data sets and serves as a pre-industrial baseline for all comparisons. Note that the annual means for a given year are calculated from December of the previous year to November of that year.

The methodology used to partition uncertainty in future projections is described in detail in a previous study [22] (hereafter HS09). We provide a brief summary of this method here. The total variance in temperature and precipitation projections is decomposed to quantify contributions from three different components: internal variability, model uncertainty, and scenario uncertainty. For both variables and for every model in the CMIP5 ensemble, we calculated anomaly time series from 1901 to 2099 relative to 1901-1930 mean. These time series were then smoothed using a 5-year averaging window and were fit with a fourth order polynomial. The internal variability contribution, which is assumed to be constant in time, is defined as the variance of the residuals from the least squares fits. The model uncertainty is based on variance in different model fits for each scenario, whereas the scenario uncertainty is the variance of the multi-model means for two scenarios. We assume that the performance of every model in simulating regional historical temperature and precipitation changes is equally credible. This is the only major difference in our approach and that discussed in HS09, which down-weighted models based on their historical performance while partitioning uncertainty in global mean temperature projections. The fractional uncertainty (at 90% confidence) is calculated from total uncertainty (sum of three contributions) and the ensemble mean projection across two RCP scenarios. The signal-to-noise ratio is the reciprocal of fractional uncertainty.

## Results

### Comparison between Threshold Crossing Times for Global and CONUS Mean Temperatures

**Fig 1:**
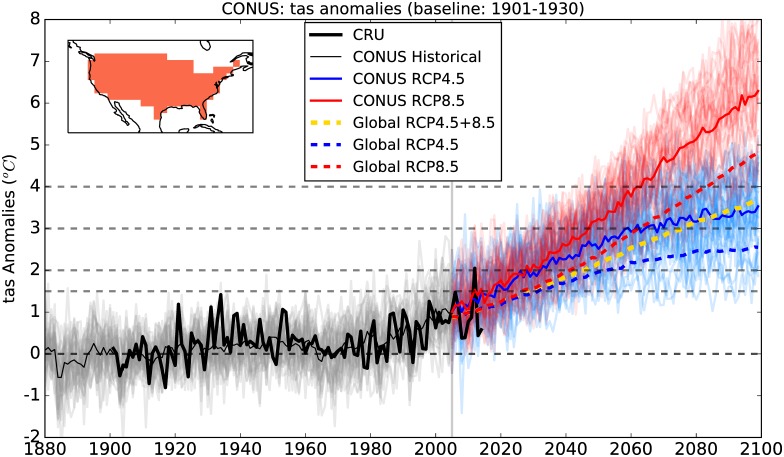
Annual mean surface air temperature anomalies relative to 1901-1930 mean for CONUS for 32 models in the CMIP5 multi-model ensemble for RCP4.5 (blue) and RCP8.5 (red). The ensemble mean and the individual model projections from 2005 to 2099 are shown by colored thin lines and the globally averaged (land+ocean) ensemble mean projections are shown by colored dotted lines. The yellow dotted line indicates the ensemble mean projections across all models and both RCPs. The dotted grey lines indicate the baseline (1901-1930) and 1.5, 2, 3, and 4°C global warming thresholds relative to the baseline. The thick black line shows the observed (CRU) temperature anomalies from 1901-2014 for CONUS. The vertical grey line marks the boundary between historical and RCP CMIP5 simulations.

The annual mean temperature anomalies relative to 1901-1930 mean (considered here to be comparable to the pre-industrial baseline) averaged over the contiguous US (CONUS) for 32 CMIP5 models and 2 RCPs are shown in Fig 1. The observed temperature evolution over the historical period is within the variability presented by the CMIP5 models. The ensemble mean projected warming over CONUS is greater than the global mean temperature increase for both RCPs (Fig 1). The ensemble mean GMAT is projected to reach 1.5°C by around 2030 for both RCPs whereas the 2°C target is reached by 2040 for RCP8.5 and by 2050 for RCP4.5. The 3°C warming is projected to be reached soon after 2060 for RCP8.5, but not before 2100 for RCP4.5. For CONUS, the ensemble mean warming (solid colored lines) reaches all these temperature thresholds at least a decade earlier than the global mean temperature (dashed colored lines).

**Fig 2:**
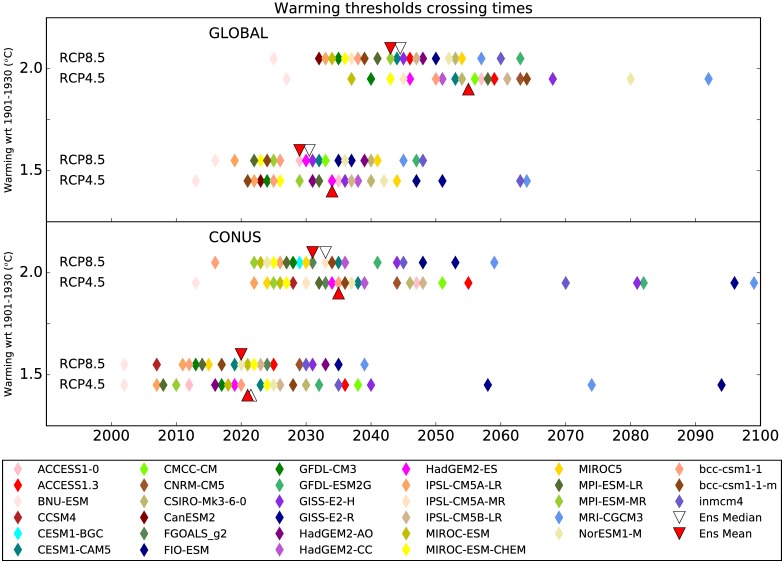
Timings for when global and CONUS 5-year mean annual mean temperatures for 32 CMIP5 models cross 1.5°C and 2°C thresholds for RCP4.5 and RCP8.5. The threshold crossing times for the ensemble mean projections are indicated by red triangles and for the ensemble median projections by open triangles. The timings for the ensemble median projections are shown only if all 32 models cross the selected threshold before 2100.

One of the important factors hampering our ability to determine the exact threshold crossing times is internal climate variability, the importance of which increases at regional scales. We calculate the threshold crossing times (TCTs) for individual models and each RCP based on 5-year moving averages instead of annual or seasonal means to minimize the effect of internal variability. The last year of the 5-year averaging window is identified as the threshold crossing time when the 5-year means for that and all the subsequent windows exceed the selected temperature threshold. This ensures that determination of the threshold crossing time is robust. Note that as a result of internal climate variability these thresholds are likely to be crossed temporarily before the TCTs identified here. The TCTs for global and CONUS mean temperatures for individual models are shown in Fig 2. Most models cross the warming thresholds earlier over CONUS than for the globe. In both RCPs, some models cross the 2°C warming threshold for CONUS as early as the 2020s, whereas some other models do not reach that mark until late in the century. Under RCP8.5, all models indicate 1.5°C warming over CONUS before 2040 and 2°C warming before 2060, but the TCTs for RCP4.5 are distributed throughout the century. Notably, the ensemble mean temperature projections over CONUS reach 2°C warming by early to mid 2030s and 1.5°C warming by early 2020s despite large differences in TCTs for individual models. The observed warming over CONUS by 2014 relative to the baseline was roughly 1.0°C but a number of models suggest it to be over 1.5°C. Such disagreements could result from the mismatch in observed and simulated variability on decadal timescales [23, 24].

### Comparison between Global, CONUS, and Regional Projections

#### Temperature Projections

**Fig 3:**
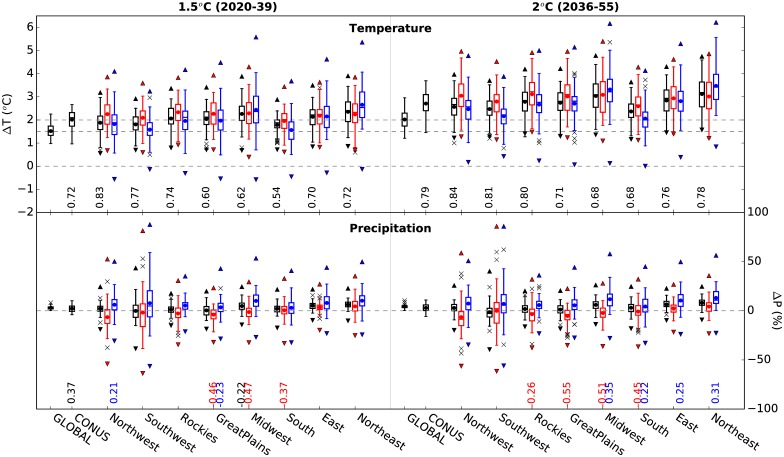
Boxplots showing the ranges in annual and seasonal temperature (top panel) and precipitation (bottom panel) projections spanned by 32 CMIP5 models for RCP4.5 and 8.5 for the globe, CONUS, and for 8 regions in CONUS. Projections are calculated for two 20-year periods when the GMAT increase is 1.5°C (left) and 2°C (right) relative to the baseline (1901-1930). The annual mean projections are in black, summer (JJA) in red and winter (DJF) in blue. Filled circles indicate the ensemble mean projections. Triangles show the 5^th^-95^th^ percentile ranges for annual and seasonal mean fields based on 32 models across 20 years and 2 RCPs. The numbers below boxplots indicate Spearman rank correlation coefficients between GMAT warming and regional mean annual warming (top panel), and between regional seasonal warming and seasonal precipitation change (bottom panel) across 64 model projections. Only statistically significant coefficients at the 90% level are shown.

The regional consequences of global warming of 1.5 and 2°C can be understood by determining the timing of when GMAT crosses these thresholds and by calculating regional projections for those times. It is evident from Fig 2 that an exact determination of when a given GMAT threshold will be crossed is not possible due to the spread in future projections. We, therefore, calculate projections for 8 regions in the US (S1 Fig) for two 20-year periods (2020-2039 and 2036-55). These two periods indicate when the mean GMAT projections calculated across all 32 models and 2 concentration pathways (RCP4.5, RCP8.5) are 1.5°C and 2°C above the baseline respectively (see yellow dotted line in Fig 1). The comparison between the ranges in global, CONUS and regional temperature and precipitation changes spanned by 64 model projections (32 models and two RCPs) are shown in Fig 3. Here, each projection is considered equally likely regardless of the model and emissions scenario. By using a large number of CMIP5 models for one medium stabilization scenario (RCP4.5) and one high emissions scenario (RCP8.5), we believe that our results span the range of uncertainties in climate change projections reasonably well.

For the 20-year period when the ensemble mean projection for GMAT is roughly 1.5°C, the individual models produce warming estimates ranging between 1°C and 2.2°C as a result of differing climate sensitivities. The ensemble mean warming for CONUS is higher (2°C) than that for GMAT and ranges from 1°C to 2.7°C. A wide range in warming at a regional scale compared to global and CONUS ranges is a result of larger climate variability and because individual models produce markedly different responses at regional and short time scales. The regional projections are strongly season-dependent; summer (JJA) warming is more pronounced in most regions than winter (DJF) warming. The only exception is the Northeast where greater warming is projected for winter. With the exception of winter warming for the South and Southwest regions, over 75% of model projections indicate higher than 1.5°C seasonal and annual mean warming for all regions by the time global warming reaches 1.5°C. For the second 20-year period corresponding to global warming of 2°C, the uncertainty in the regional projections is higher. The lower bounds of temperature projections in Fig 3 typically represent how low climate sensitivity models respond to RCP4.5 whereas the higher bounds are determined by the responses of high climate sensitivity models to RCP8.5. Since the low sensitivity models indicate very little warming globally as well as regionally throughout the century, the lower bounds of projections are comparable across regions. Additionally, RCP4.5 is a stabilization scenario [25, 26] resulting in a lower rate of warming in the latter half of the century than the next few decades [17]. The largest differences between global and regional projections can be seen at the higher end of regional projections. The ensemble mean and median warming estimate for all regions in the US are higher than those for the globe, indicating that the enhanced warming at regional scales is projected by most models in the CMIP5 ensemble. Overall, the models that project greater (smaller) warming at a global scale also project greater (smaller) warming at regional scales as indicated by high Spearman rank correlations between the global and regional warming across all model projections (see Fig 3). Among the regions examined, the Northeast and the Midwest are projected to warm most with over 2.5°C warming in both seasons when the GMAT increase is 2°C. While the 20-year mean projections describe the overall effect of global warming on a region, they mask the interannual variations that are important for impacts assessment. Year-to-year estimates of regional temperature projections over the two 20-year periods show significant variation (see 5^th^-95^th^ percentile ranges in Fig 3), particularly in winter, compared to the ranges spanned by 20-year mean projections. This result highlights the influence of climate variability at regional scales on our results. Nonetheless, the regional projections for the GMAT increase of 2°C can be clearly distinguished from those for the GMAT increase of 1.5°C.

#### Precipitation Projections

Similar analysis for precipitation shows that all models indicate an increase in mean annual global precipitation and 75% of the models indicate an increase in mean annual CONUS precipitation for both global warming thresholds (Fig 3 and S2 Fig). The regional projections, however, are highly uncertain for both 20-year periods (2020-2039 and 2036-55), and show no significant differences in ensemble spread for two global warming thresholds. Previous studies for the late 21^st^ century projections based on CMIP3 and CMIP5 ensemble means note an increase in winter precipitation over most of the CONUS with the exception of south-central US and a significant decrease in summer precipitation in parts of central and northwest US [16, 17, 27]. These studies also note a lack of model agreement in the magnitude and direction of precipitation change over most CONUS in summer. In general, the CMIP5 models project a decrease in precipitation in summer and an increase in winter, but the full range spans both wetter and drier projections. The exceptions to this are winter precipitation projections for the eastern US (that includes the Northeast) and the Midwest where most CMIP5 models indicate a wetter future compared to the baseline. Additionally, 75% of the models also indicate wetter summers in the Northeast during both 20-year periods leading to an overall increase in annual mean precipitation. The changes over the Northeast are in agreement with a previous study [28] that examined projected precipitation changes in dynamical downscaling experiments. Since the projections are presented as a percentage change, the relatively dry regions such as the Southwest and the Rockies have very large spread in their precipitation projections across the ensemble. A robust drying pattern has been identified for the Southwest in spring [16, 29] (not shown), which leads to an overall decrease in annual precipitation noted in Fig 3. Also, a lack of agreement amongst models on the direction of change should be treated with caution as it does not necessarily imply a lack of climate change signal [30]. And for arid regions of the Southwest, a few percent decrease in precipitation combined with increasing temperatures can lead to significant reduction in water availability [31]. Large negative Spearman rank correlations between temperature and precipitation changes in summer in the South, Midwest, and Great Plains regions suggest that the models with higher warming project stronger drying in summer in these regions. On the other hand, in winter, models that project more warming also tend to indicate a higher increase in precipitation in the Midwest and the East. But unlike temperature, large variability on interannual timescales makes it difficult to determine the effect of additional GMAT increase of 0.5°C on regional precipitation.

### Consensus among Models

Uncertainty in climate change projections is described in two different ways in Figs 2 and 3. The former shows uncertainty in the timing of a given climate change outcome, whereas the latter shows uncertainty in the magnitude of change for a given period. The timing of when CONUS mean annual temperature will cross the 1.5°C and 2°C thresholds differs substantially between models and greenhouse gas concentrations pathways (Fig 2) and similar analyses for regional temperatures show even more disagreement. Alternatively, Fig 3 provides a regional perspective for different global warming thresholds, but the ranges of regional projections are wide and may pose difficulties for planners and decision-makers. This information can, however, be expressed in terms of the percentage of models crossing a particular global warming threshold at any given time to reveal consensus amidst uncertainty.

**Fig 4:**
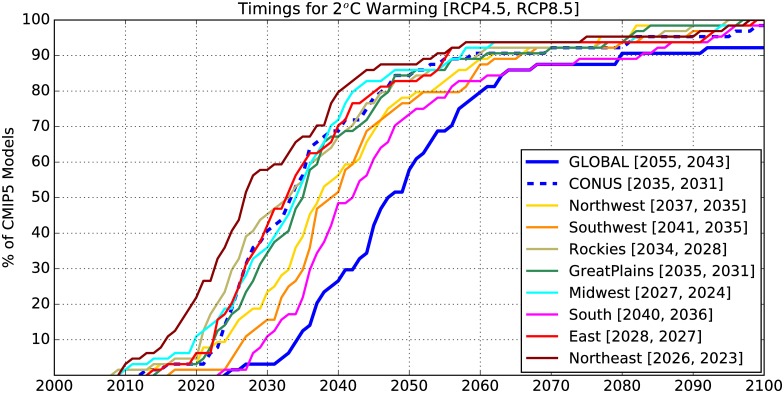
A comparison between 2°C threshold crossing times for global, CONUS and regional 5-year mean annual mean temperatures shown in terms of the percentage of climate models out of 64 total projections (32 RCP4.5 + 32 RCP8.5) that cross the 2°C threshold at a given time until 2099. The numbers in square brackets indicate when the multi-model ensemble mean crosses the 2°C threshold for a given region for two RCPs [RCP4.5, RCP8.5].

For the regional warming threshold of 2°C, the variation in TCTs resulting from different model responses is much larger than that resulting from different scenarios. The regional 2°C TCTs for the ensemble mean projections for RCP4.5 lag behind those for RCP8.5 only by about 1 to 6 years across the US regions. Therefore, in Fig 4 we have combined all CMIP5 projections (a total of 64; 32 each for RCP4.5 and RCP8.5) to show the fraction crossing a 2°C threshold globally, for CONUS, and for eight US regions (cf. S1 Fig) at any given time in the 21^st^ century. It is clear from Fig 4 that the CONUS and regional mean annual temperatures cross the 2°C warming threshold well before GMAT. Regions within the US show different warming rates with mean annual temperatures in the South, Southwest, Great Plains rising slower than that for CONUS. The Northeast is projected to warm the fastest whereas the South is slowest (although still faster than the global mean). The Northeast is projected to cross the 2°C threshold about 15 years earlier than the South and about two decades before GMAT. For instance, about 80% of the projections indicate 2°C global warming by 2060 whereas the same percentage of projections cross the 2°C threshold in the Northeast by 2040. Additionally, all models project faster warming in the Northeast and the eastern US than the South and the Southwest, which indicates that there is an agreement among models with regard to the pattern of differential warming across the US even though they disagree on the threshold crossing times. Previous studies [16, 17] do not discuss differential warming across the country as this aspect of warming does not naturally emerge from the interpretation of multi-model and multi-decade mean maps typically used to present climate change information. In contrast, Figs 3 and 4 clearly demonstrate that there are variations in the rate of warming across the US and indicate what the model consensus behind differential warming is.

**Fig 5:**
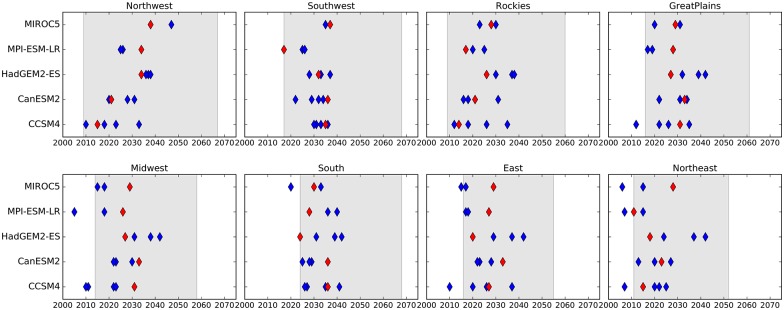
Impact of internal climate variability on the threshold crossing times. The regional 2°C TCTs are calculated based on regional 5-year mean annual mean temperatures for RCP8.5 for 5 CMIP5 models with initial conditions ensembles. The red symbol indicates TCTs for one realization of the model included in all analyses. The blue symbols show TCTs for different members of the initial conditions ensembles. Gray shaded area indicates the spread in regional TCTs based on all 32 models, each with one realization.

The regional TCTs discussed earlier are calculated for a single realization of every model, and by design, do not fully capture the impact of internal variability. The 5-year running means damp interannual variations, and provide reasonably accurate TCTs for a given realization of a climate model. But another realization of the same model with a different flavor of internal variability may lead to different TCTs. Several models participating in the CMIP5 activity submitted multiple simulations that were initialized using different but realistic initial conditions (IC) in an effort to sample internal climate variability [18]. We use 5 CMIP5 models with multiple initial conditions simulations to investigate how internal variability affects regional TCTs. Fig 5 shows that the regional 2°C TCTs for RCP8.5 can be different by a few years to two decades as a result of the unpredictable nature of internal climate variations. All models show significant spread in TCTs across the IC ensemble for northern regions such as the Northeast and the Midwest, but the spread is comparatively smaller for regions in the southern US (South, Southwest). This uncertainty in TCTs due to internal variability is, however, smaller than the spread in TCTs arising from using different models (indicated by gray shading in Fig 5) for all regions. So we conclude that while the TCTs based on 5-year means might be affected by the nature of decadal variability in individual models, the overall spread in TCTs is mainly due to differing sensitivities of these models to future changes in radiative forcing.

**Fig 6:**
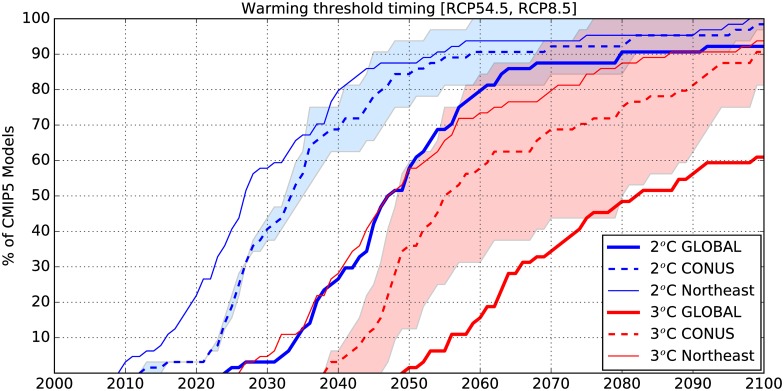
Same as Fig 4 but showing comparisons between 2°C and 3°C threshold crossing times for the globe, CONUS and the Northeast US 5-year mean annual mean temperatures. The left edges of the shaded areas show the TCTs for RCP8.5 and the right edges for RCP4.5 for CONUS.

The impact of two different RCPs on threshold crossing times is shown in Fig 6. By 2060, about 90% of the CMIP5 projections indicate at least 2°C warming over CONUS. For RCP8.5, all models cross this threshold by 2060 globally as well as regionally (S3 Fig), whereas for RCP4.5 only 4 models (out of 32) cross the 2°C warming threshold after 2060. Since the differences between RCPs are larger at longer lead times, models disagree more on crossing times for 3°C than for 2°C. Nonetheless, over 90% of the projections indicate at least 2°C warming and about 60% indicate 3°C warming over CONUS by 2060. Since the Northeast is projected to cross the 2°C threshold about 20 years earlier than GMAT, it is projected to warm by 3°C (Fig 6, solid thin red line) relative to the baseline by the time GMAT reaches 2°C (solid thick blue line).

### Partitioning Uncertainty

Uncertainty in climate change projections is typically partitioned into three components: internal variability (IV), model uncertainty and scenario uncertainty [22]. Partitioning uncertainty in this way has two distinct advantages: it helps in the design of new modeling strategies to reduce uncertainty [22, 32] and it informs the use of climate data in impacts assessment by providing information on the relative importance of different uncertainties as a function of time. Here, we focus on the second aspect. In particular, we are interested in the signal-to-noise (S/N) ratio—the strength of the climate change signal relative to the ‘noise’ resulting from different sources of uncertainty in model projections. While the consensus in model results as described in the previous section is generally used to support decisions, that argument can be strengthened further if the S/N ratio is high (or at least above 1) for a variable, region and timescales of interest. We partition uncertainty in regional temperature and precipitation projections and calculate the S/N ratios following HS09 method [22] (see Data and Methods).

**Fig 7:**
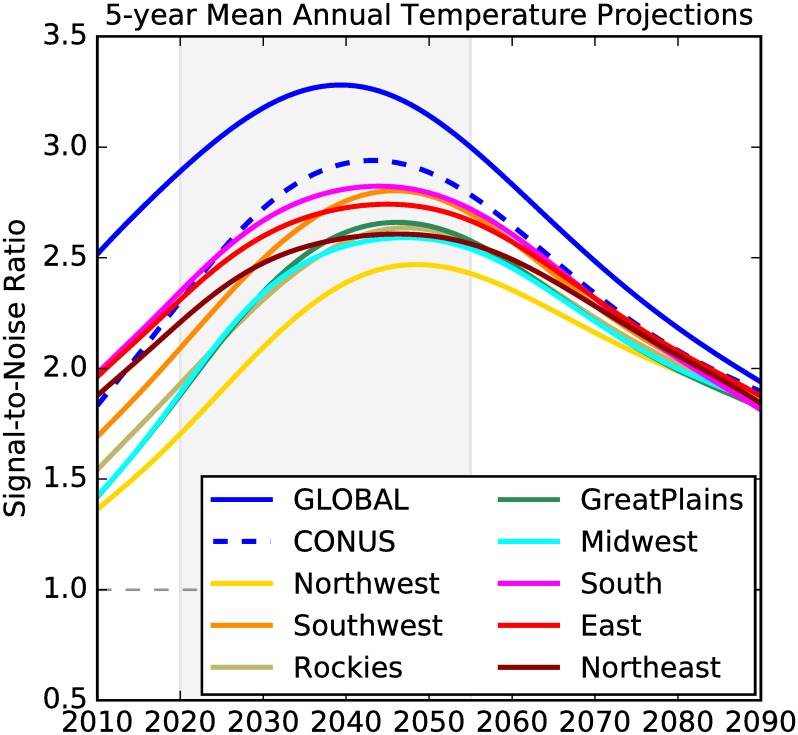
Signal-to-noise ratio for 5-year mean annual mean global, CONUS, and regional temperatures. Grey shading indicates the period during which the mean GMAT projections calculated across all 32 models and 2 RCPs are 1.5°C and 2°C above the baseline.

Fig 7 shows a comparison between global, CONUS, and regional S/N ratios. Since climate variability is typically larger at smaller spatial scales, the regional S/N ratios are smaller than that for CONUS, which in turn is smaller than S/N for the global mean temperature. Nevertheless, the regional S/N values remain above 1 throughout the century and peak during the two 20-year periods from 2020 to 2055 when the ensemble mean GMAT is projected to cross the 1.5 and 2°C thresholds. This suggests that the regional warming signal in most of the US is projected to be substantially greater than the uncertainty in projections produced by the CMIP5 ensemble, raising confidence in the potential usefulness of these projections for planners. While the S/N ratios shown in Fig 7 are based on 5-year running means of annual mean temperature time series, the S/N ratios are above 1 for annual mean temperatures as well. Differences among the S/N ratios across different regions in the US could also arise due to variations in the size of the regions considered here. For precipitation projections, however, the S/N is below 1 for annual, 5-year and decadal mean precipitation (not shown). There results are broadly consistent with previous studies [22, 32].

**Fig 8:**
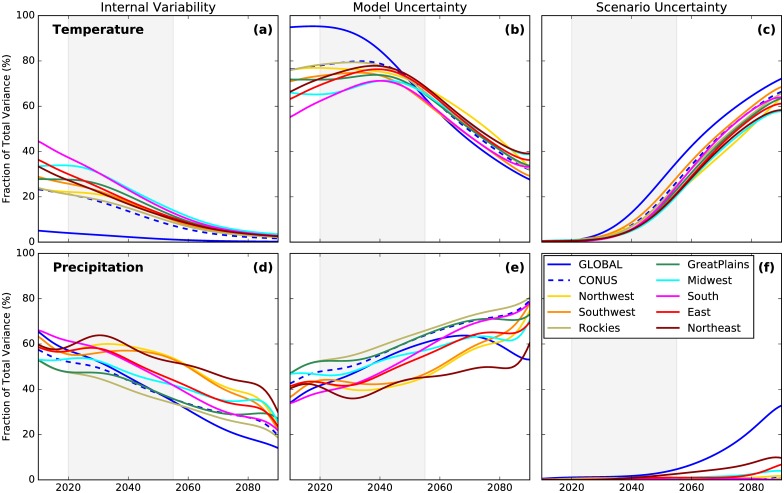
Importance of three sources of uncertainty in the projections of 5-year mean annual mean precipitation. Uncertainty is partitioned based on the methodology described in HS09. Grey shading indicates the period during which the mean GMAT projections calculated across all 32 models and 2 RCPs are 1.5°C and 2°C above the baseline.

The contribution of three components to the total uncertainty in 5-year mean annual mean temperature and precipitation projections is shown in Fig 8. The importance of internal variability at regional scales and for near-term projections (next 2-3 decades) and the dominance of model uncertainty throughout the century are clearly evident for all regions in the US for both variables. The contribution of model uncertainty decreases slightly in the second half of the century as scenario uncertainty becomes important for temperature projections. For regional precipitation, however, the contribution of model uncertainty increases with time with the diminishing importance of internal variability. Scenario uncertainty becomes important for global mean precipitation (at the expense of model uncertainty) during the latter half of this century, but remains mostly unimportant at regional scales considered in this study. For two regions, the East and the Northeast US there is an indication that scenario uncertainty may contribute to the total uncertainty in the last couple of decades of this century. Also, the impact of spatial averaging is clearly evident wherein the internal variability contribution is more important for smaller regions. The contribution of internal variability to the total uncertainty diminishes if these calculations are performed for decadal mean precipitation and increases at the expense of model uncertainty for annual mean precipitation (not shown). Time averaging, however, does not affect the contribution of scenario uncertainty to the total uncertainty. Overall, for the period when the GMAT crosses 1.5 and 2°C, the uncertainty in regional temperature and precipitation projections is mostly dominated by internal climate variability and model diversity.

## Conclusions and Discussion

Assessing the impacts of a changing climate requires region-specific climate change information with guidance on associated uncertainties. In this study, we discuss the implications of global temperature targets set in Paris for the timing and magnitude of regional temperature and precipitation changes in the US using the CMIP5 multi-model ensemble. This threshold-based approach identifies timings associated with regional climatic changes, and clearly demonstrates how warming rates are projected to differ across the country. These aspects cannot be easily deduced from the traditional approach in which climate projections are presented in the form of a multi-model mean for a certain 20-30 year time period in the future. We also highlight consensus and disagreements in model projections, and the relative importance of different types of uncertainty in these projections as a function of time. Together, these factors provide information that can be useful to effectively communicate the consequence of the agreement reached in Paris to the regional stakeholders.

All regions in the contiguous United States are projected to cross the 2°C warming threshold about 10-20 years earlier than the global mean annual temperature. While there is a large spread in TCTs across all regions, 75% to 90% of the models reach 2°C warming by 2050 for every region in the US. We believe that our estimates of TCTs based on 5-year means of annual mean temperatures may be conservative since we require that all the subsequent 5-year means beyond the identified threshold crossing time exceed the selected temperature threshold. The unpredictable nature of internal climate variability could advance or delay TCTs by a few years to a couple of decades regionally as demonstrated using the initials conditions ensemble. But this uncertainty in regional TCTs is smaller than the spread arising from using different models and two different scenarios. The consequence of large climate variability at regional scales, however, suggests that it may prove difficult to distinguish between the consequences of global warming of 1.5°C and 2°C for regional changes. Note also that the selected temperature thresholds may be temporarily crossed earlier than the identified TCTs as a result of climate variability on seasonal and interannual timescales. In fact, regional warming of 2°C relative to the baseline has already been observed for some years for all regions in the US except for the South (S4 Fig). A cooling trend in the southeast US, the so called “warming hole”, in the second half of the twentieth century was not captured by CMIP5 models [33]. Although this cooling trend has disappeared since the 1990s [34], the CMIP5 models have overestimated warming in the region over the last two decades (S4 Fig), which suggests that the actual TCTs for the South could be later than those presented here. On the other hand, the eastern US is projected to reach the 2°C target in the 2020s regardless of the scenarios. This result seems realistic given that the Paris Agreement will not come into force until 2020 [1] and that the model projections for the region match the current trends of regional warming well [35]. Importantly, the timescales associated with the eventual 1.5 and 2°C warming are long, requiring rapid development and deployment of efficient technologies throughout the 21^st^ century [9]. Consequently, we believe that the near-term regional projections discussed here may indeed be realized regardless of what greenhouse gas concentrations pathway the world takes to meet the COP21 temperature goals.

The effect of different global temperature targets on climate extremes has been a focus of a number of previous studies. The global occurrence of temperature and precipitation extremes has been shown to increase non-linearly with global mean temperature [7, 36] leading to large increases in high-impact extreme events for every degree increase in temperature. While the observed trends in the temperature extremes are within the bounds of natural variability [37], results based on CMIP3 and CMIP5 models indicate a substantial increase in summer temperature extremes in the US before global warming reaches 2°C [38]. The extreme high temperatures over CONUS are projected to increase linearly with GMAT, but at a slightly faster rate [6]. While such trends in extremes have been noted, overall, the model projections of regional climate extremes remain highly uncertain due to differing model responses and large climate variability.

There are a number of caveats in the HS09 method [22] used to partition uncertainty in model projections such as the use of only two RCPs, and the constancy of internal variability estimates in time. The CMIP5 multi-model ensemble used in this study samples the structural diversity in model formulation, but was not designed for systematic exploration of uncertainties, and therefore may not span the full range of outcomes in climate projections [39]. Additionally, the use of one realization for every model is inadequate to capture the effect of internal variability that plays a significant role in driving regional changes from years to decades. While these shortcomings are not expected to affect the conclusions drawn in this work, we suggest that the uncertainty estimates be treated as reasonable guidelines. Overall, decomposing uncertainty in regional precipitation projections indicates the importance of internal variability in the short-term, model uncertainty in the long-term, and very little to no contribution of scenario uncertainty throughout the 21^st^ century. In the absence of reliable constraints on future precipitation projections [40], assessment of regional impacts should be based on climate information obtained from a large number of models with diverse outcomes instead of putting too much emphasis on using different scenarios for a handful of models. Note that the uncertainty partitioning is relative in nature. If future generations of models reduce contributions from internal variability and model uncertainty substantially, studying precipitation response to different scenarios may become important. This, however, is unlikely given that the internal climate variations over CONUS remain large and highly unpredictable over the next 20-50 years [41, 42].

## Supporting Information

S1 Text List of 32 CMIP5 models included in the analysis. (PDF)

S1 File Temperature data files. Annual and seasonal mean anomalies of surface air temperature for 1871-2099 relative to 1901-1930 mean for 32 CMIP5 models for RCP4.5 and RCP8.5 for the contiguous United States.
(ZIP)

S2 File Precipitation data files. Annual and seasonal mean anomalies of precipitation for 1871-2099 relative to 1901-1930 mean for 32 CMIP5 models for RCP4.5 and RCP8.5 for the contiguous United States.
(ZIP)

S1 Fig Masks used to calculate regional projection. The masks are at 2.5°×2.5° resolution and created based on ‘Bukovsky regions’ [43]. The region definitions in terms of Bukovsky regions are as follows: Northwest (NW): PacificNW+GreatBasin, Southwest (SW): PacificSW+Southwest, Rockies (R): NRockies+SRockies, GreatPlains (PL): CPlains+SPlains+Central, Midwest (MW), South (S), East (E), Northeast (NE). Some regions are created by merging original Bukovsky regions to avoid using too few grid-boxes in spatial and temporal averaging. Note that regions NorthAtlantic and Plains are sub-regions of East and Central respectively.
(TIFF)

S2 Fig Same as Fig 1 but for annual mean precipitation anomalies relative to 1901-1930 mean for CONUS for 32 models in the CMIP5 multi-model ensemble for RCP4.5 (blue) and RCP8.5 (red). The ensemble mean and the individual model projections from 2005 to 2099 are shown by colored thin lines and the globally averaged (land+ocean) ensemble mean projections are shown by colored dotted lines. The yellow dotted line indicates the ensemble mean projections across all models and both RCPs. The dotted grey lines indicate the baseline (1901-1930). The thick black line shows the observed (UDel) precipitation anomalies from 1901-2014 for CONUS. The vertical grey line marks the boundary between historical and RCP CMIP5 simulations.
(TIFF)

S3 Fig Same as Fig 4 but for projections based on 32 CMIP5 models for RCP8.5. (TIFF)

S4 Fig Same as Fig 1, but for 8 regions in the contiguous US shown in S1 Fig. Annual mean surface air temperature anomalies relative to 1901-1930 mean for 32 models in the CMIP5 multi-model ensemble for RCP4.5 (blue) and RCP8.5 (red). The ensemble mean and the individual model projections from 2005 to 2099 are shown by colored thin lines and the globally averaged (land+ocean) ensemble mean projections are shown by colored dotted lines. The dotted grey lines indicate the baseline (1901-1930) and 1.5, 2, 3, and 4°C warming thresholds. The thick black line shows the observed (CRU) temperature anomalies from 1901-2014 for CONUS. The vertical grey line marks the boundary between historical and RCP CMIP5 simulations.
(TIFF)
