# Supplementary material for: Consequences of Global Warming of 1.5 °C and 2 °C for Regional Temperature and Precipitation Changes in the Contiguous United States
Source: PLoS One. 2017 Jan 11;12(1):e0168697. doi: 10.1371/journal.pone.0168697 (PMC5226673; doi:10.1371/journal.pone.0168697)
Supplement: S1 Text — (PDF) [file pone.0168697.s001.pdf]

List of CMIP5 models:

ACCESS1-0, ACCESS1.3, BNU-ESM, CCSM4, CESM1-BGC, CESM1-CAM5, CMCC-CM, CNRM-CM5, CSIRO-Mk3-6-0, CanESM2, FGOALS_g2, FIO-ESM, GFDL-CM3, GFDL-ESM2G, GISS-E2-H, GISS-E2-R, HadGEM2-AO, HadGEM2-CC, HadGEM2-ES, IPSL-CM5A-LR, IPSL-CM5A-MR, IPSL-CM5B-LR, MIROC-ESM, MIROC-ESM-CHEM, MIROC5, MPI-ESM-LR, MPI-ESM-MR, MRI-CGCM3, NorESM1-M, bcc-csm1-1, bcc-csm1-1-m, inmcm4.
