# Supplementary figures and images for: Consequences of Global Warming of 1.5 °C and 2 °C for Regional Temperature and Precipitation Changes in the Contiguous United States
Source: PLoS One. 2017 Jan 11;12(1):e0168697. doi: 10.1371/journal.pone.0168697 (PMC5226673; doi:10.1371/journal.pone.0168697)

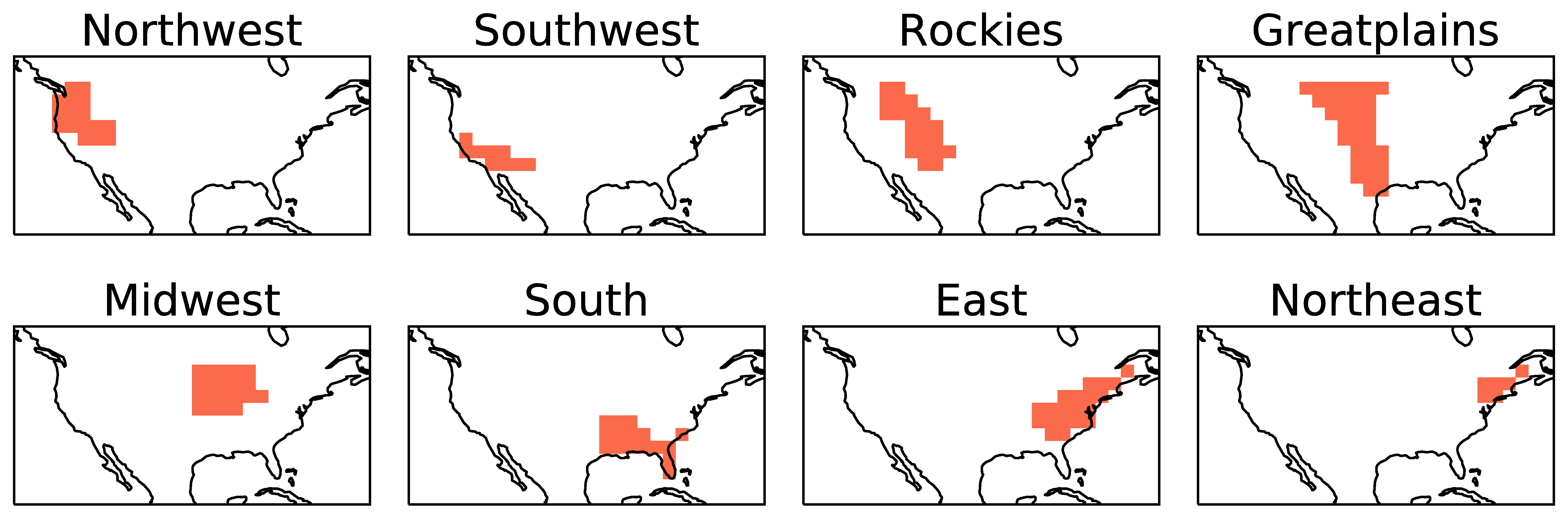

Supplement: S1 Fig — The masks are at 2.5°×2.5° resolution and created based on ‘Bukovsky regions’ [43]. The region definitions in terms of Bukovsky regions are as follows: Northwest (NW): PacificNW+GreatBasin, Southwest (SW): PacificSW+Southwest, Rockies (R): NRockies+SRockies, GreatPlains (PL): CPlains+SPlains+Central, Midwest (MW), South (S), East (E), Northeast (NE). Some regions are created by merging original Bukovsky regions to avoid using too few grid-boxes in spatial and temporal averaging. Note that regions NorthAtlantic and Plains are sub-regions of East and Central respectively. (TIFF) [file pone.0168697.s004.tiff]

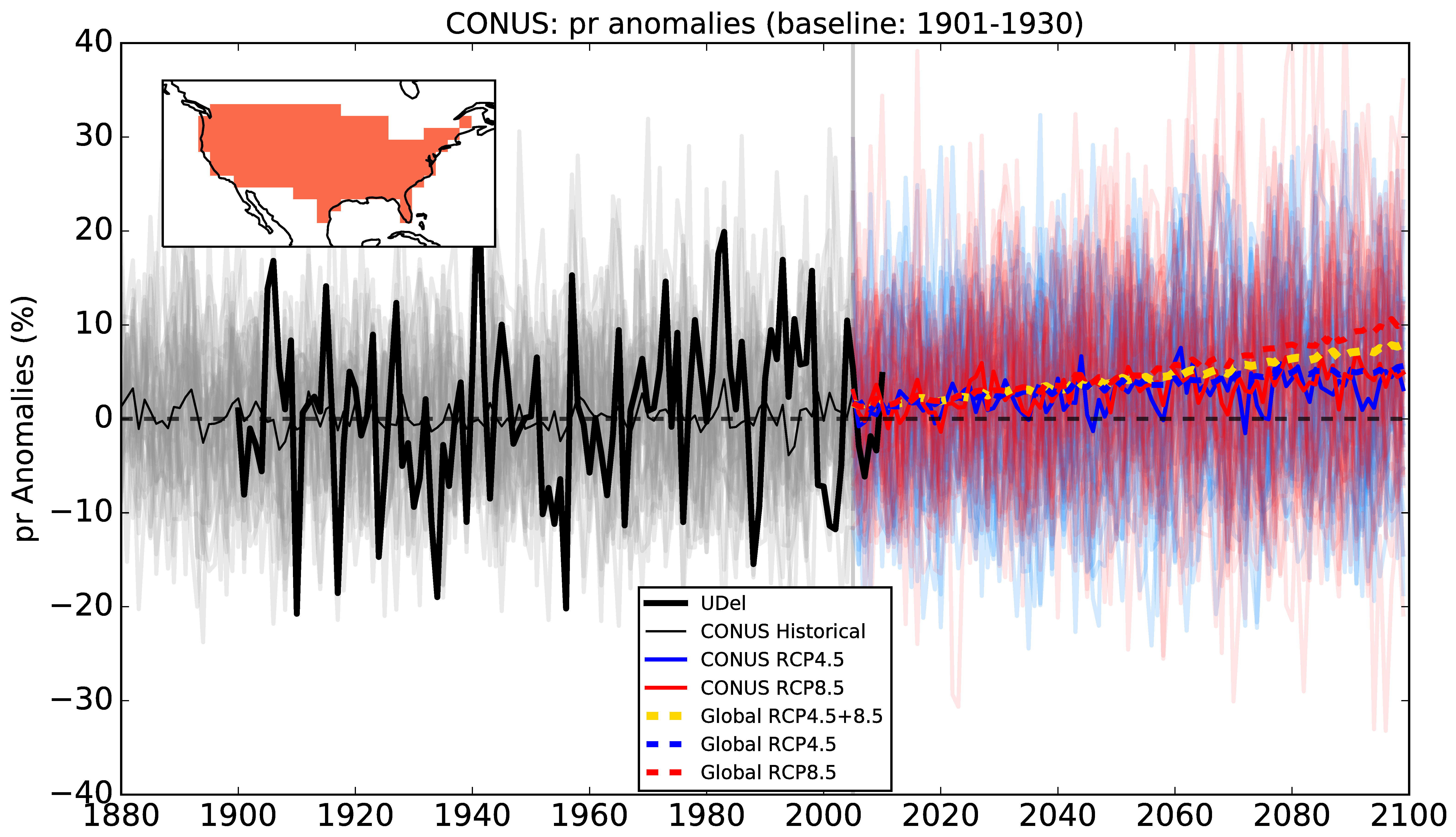

Supplement: S2 Fig — The ensemble mean and the individual model projections from 2005 to 2099 are shown by colored thin lines and the globally averaged (land+ocean) ensemble mean projections are shown by colored dotted lines. The yellow dotted line indicates the ensemble mean projections across all models and both RCPs. The dotted grey lines indicate the baseline (1901-1930). The thick black line shows the observed (UDel) precipitation anomalies from 1901-2014 for CONUS. The vertical grey line marks the boundary between historical and RCP CMIP5 simulations. (TIFF) [file pone.0168697.s005.tiff]

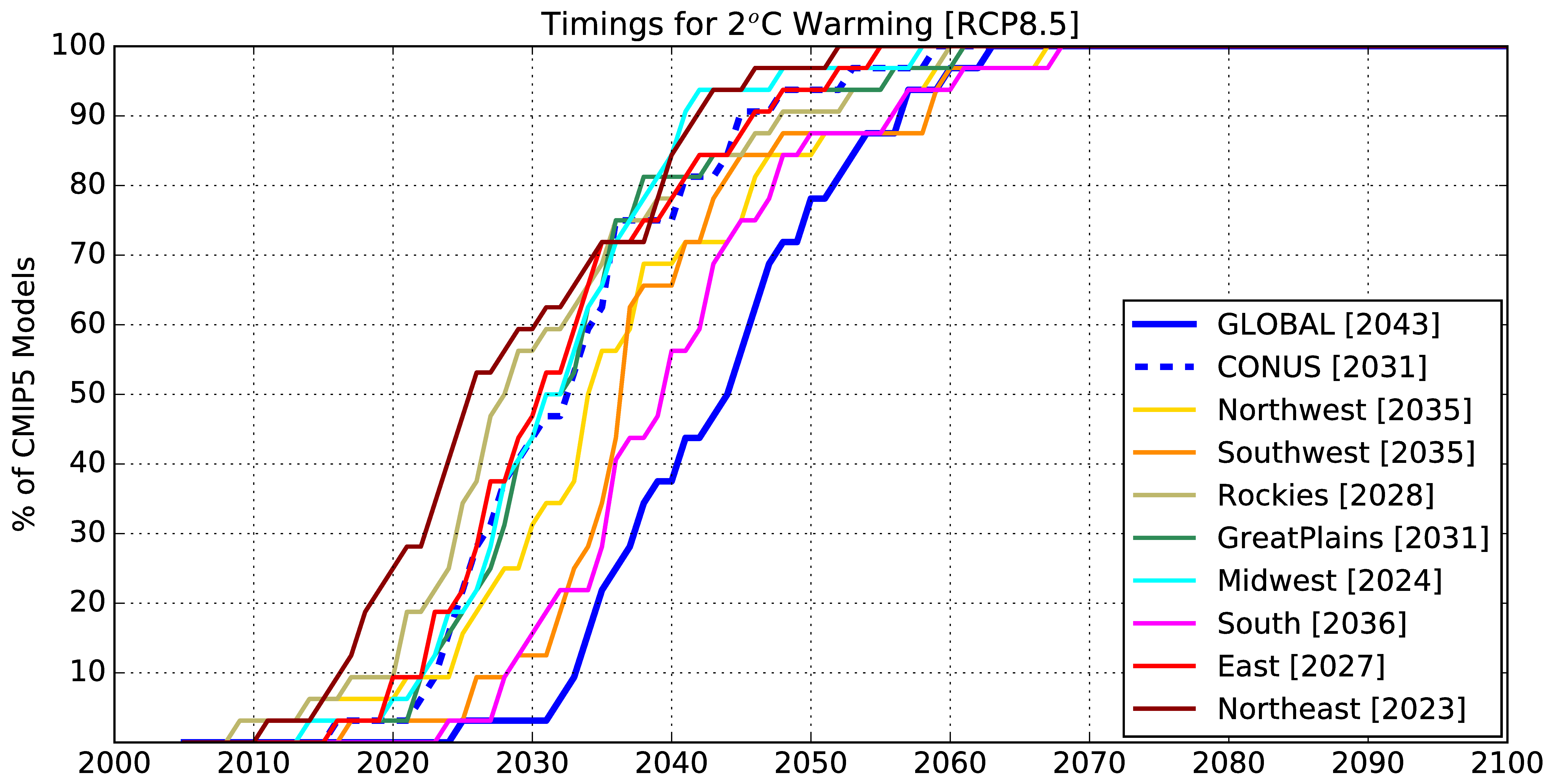

Supplement: S3 Fig — (TIFF) [file pone.0168697.s006.tiff]

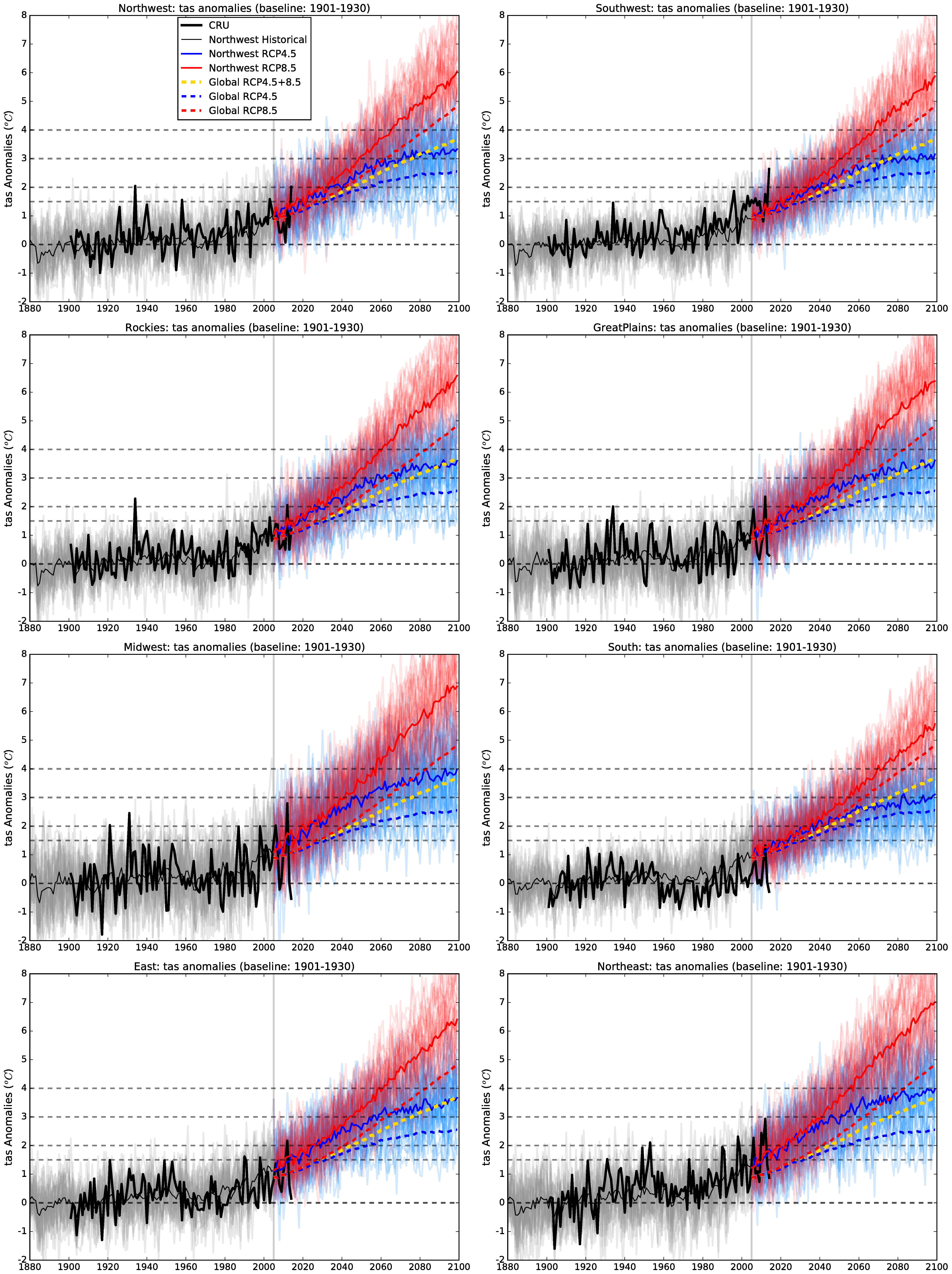

Supplement: S4 Fig — Annual mean surface air temperature anomalies relative to 1901-1930 mean for 32 models in the CMIP5 multi-model ensemble for RCP4.5 (blue) and RCP8.5 (red). The ensemble mean and the individual model projections from 2005 to 2099 are shown by colored thin lines and the globally averaged (land+ocean) ensemble mean projections are shown by colored dotted lines. The dotted grey lines indicate the baseline (1901-1930) and 1.5, 2, 3, and 4°C warming thresholds. The thick black line shows the observed (CRU) temperature anomalies from 1901-2014 for CONUS. The vertical grey line marks the boundary between historical and RCP CMIP5 simulations. (TIFF) [file pone.0168697.s007.tiff]
